# Minimally invasive perventricular device closure of doubly committed sub-arterial ventricular septal defects: single center long-term follow-up results

**DOI:** 10.1186/s13019-015-0326-6

**Published:** 2015-09-15

**Authors:** Shu Zhang, Da Zhu, Qi An, Hong Tang, Dajiang Li, Ke Lin

**Affiliations:** 1 Pediatric Heart Center and Department of Emergency Medicine, West China Hospital, Sichuan University, Chengdu, China; 2 Pediatric Heart Center and Department of Cardiovascular Surgery, West China hospital, Sichuan University, Chengdu, Sichuan 610041 China; 3 Pediatric Heart Center and Department of Cardiology, West China hospital, Sichuan University, Chengdu, China

**Keywords:** Congenital heart defect, Doubly committed sub-arterial ventricular septal defect, Hybrid perventricular device closure

## Abstract

**Background:**

To evaluate the long-term safety and efficacy of using perventricular device closure in treating selected patient with doubly committed sub-arterial ventricular septal defect (VSD)

**Methods:**

During July 2007 and April 2011, 86 patients with doubly committed subarterial VSD who met the inclusion criteria were enrolled in this study. Perventricular closure was attempted using a unique design eccentric device under the guidance of transesophageal echocardiography. Complications such as residual shunt, arrhythmia, valve regurgitation were all recorded in postoperative period and during follow-up. Multiple logistic regression analysis was performed to study risk factors for procedure failure and complications.

**Result:**

Perventricular device closure was successfully done in 75 patients (87.2 %) with mean age 7.0 ± 7.0 years old, VSD size 4.8 ± 1.5 mm and device size 6.7 ± 1.7 mm. Complete closure rate was achieved in 94.7 % at discharge and 96 % during follow-up. No severe complications such as device embolism, significant arrhythmia, left ventricular outflow tract obstruction as well as obvious valve regurgitation were noted during follow-up (Mean 4.5 ± 1.5 years). Procedure induced trivial-mild grade aortic valve regurgitation (AR) was noted in 16 (21.3 %) patients at discharge while 8 of them resolved during follow-up. Multivariable analysis revealed that procedure-induced AR was associated device diameter to patients’ weight (OR = 12.3 95 % CI 1.5- 99.2). Perventricular device closure was failed in 11 patients, preoperative aortic valve prolapse was the major risk factor for failure of the procedure (OR = 65 95 % CI 7.5- 564.1).

**Conclusion:**

Perventricular closure of doubly committed subarterial VSDs appears to be a safe and effective minimally invasive treatment option in selected patients with good long-term outcomes.

**Clinical trial registration:**

Unique Identifier: ChiCTR-TNC-00000203.

## Background

Doubly committed sub-arterial ventricular septal defect (VSD) is a unique type of VSDs located just beneath the aortic and pulmonary valve, accounting for approximately 5 % of all VSDs [1, 2]. Due to its unique location, this type of VSD is characterized by low tendency for spontaneous closure and progressive aortic valve leaflets prolapse as well as insufficiency [3, 4]. Traditional open-heart surgery is the mainstream treatment option, but this method is associated with complications of cardiopulmonary bypass such as systemic inflammation response, myocardium dysfunction, as well as blood transfusion especially for pediatric patients [5–7]. Meanwhile, this type of VSD is still a contraindication as for transcatheter device closure due to difficulty in delivery track building as well as adjusting the position of eccentric device during deployment.

In recent years, minimally invasive hybrid perventricular device closure has been introduced as an alternative treatment option for selected patients with doubly committed sub-arterial VSD [8–12]. Previous report has revealed the safety and efficacy of this minimal invasive technique in compared with traditional open-heart surgery [11]. The purpose of this study is to evaluate the long-term safety and efficacy of using perventricular device closure in treating selected patient with doubly committed sub-arterial ventricular septal defect and further illustrate the indication as well as contraindication as for this new technique.

## Method

### Patient selection

The Chinese Clinical Trial Registry and Ethics Committee of our hospital approved this study. Patients with doubly committed sub-arterial VSDs admitted to our hospital for surgical intervention between July 2007 and April 2011 were enrolled in this study. Including criteria were 1) Confirmed diagnosis of doubly committed sub-arterial VSD by transthoracic echocardiogram (TTE); 2) VSD diameter from 3-10 mm (less than 10 mm); 3) without pulmonary hypertension as well as other congenital heart defect. 4) Without aortic valve prolapse or only mild degree prolapse. Exclusion criteria were: 1) More than trivial grade aortic regurgitation 2) Obvious aortic valve prolapse; 3) Complicated with other congenital heart defect; 4) VSD ≥ 10 mm; 5) Preoperative frequent arrhythmias; 6) Confirmed pulmonary hypertension. The informed consents were obtained.

### Perventricular closure technique (Fig. 1)

The technique of hybrid perventricular closure of doubly committed sub-arterial VSD has been described previously with some modification [10, 11]. Transesophageal echocardiography (TEE) (Philips iE33, Andover MA with adult X7-2 t or pediatric S7-2 t probes) was used after induction of general anesthesia. The diameters of the VSD were measured through TEE as well as fluoroscopy if available. All heart valves, especially the aortic valve, were carefully checked for pre-existing regurgitation. An eccentric occlude device (Shanghai Shape Memory Alloy Co. Ltd, China) about 1–2 mm larger in size than the diameter of VSD was chosen and prepared along with the delivery system in the standard fashion. The flange of the right ventricular disc in the eccentric side are 2 mm wide but the flange of the left ventricular disc facing the aortic valve is 0 mm so as to prevent impingement of the aortic valve. The opposite flange at the same disk is 5 mm long with a metallic mark on its edge to indicate the orientation. The entire delivery system consisted of flexible guide-wire, double-lumen delivery sheath as well as loading sheath.

**Fig. 1:**
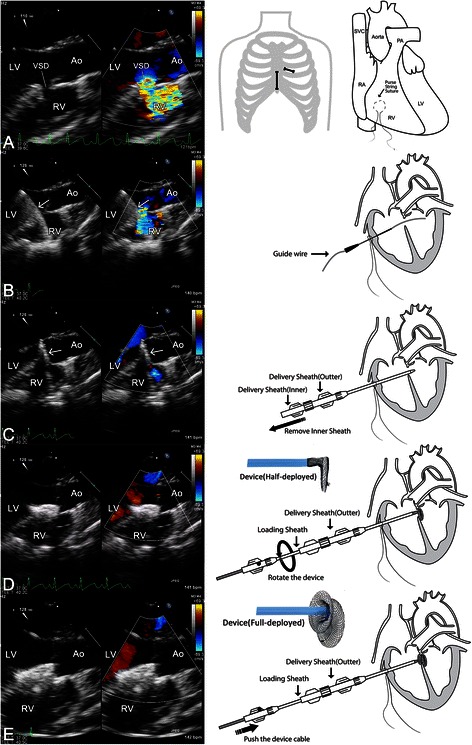
Sequential TEE as well as illustration images of perventricular device closure of doubly committed subarterial VSD. Panel **a**: TEE showed the VSD, surgical incision was made and a pledgetted mattress suture placed around the chosen puncture site in right ventricle. Panel **b**: A 20-G needle was inserted into the right ventricular cavity and a flexible 0.035-inch guidewire was introduced into the left ventricle through the defect. Panel **c**: A double-lumen delivery sheath was advanced into the left ventricle over the guidewire, and then the inner sheath was removed. Panel **d**: Occlude device was delivered through the prepared loading sheath and the delivery sheath. The left ventricular disc was deployed first by pushing the cable forward and then rotated until the eccentric side of the left disc toward the opposite side of the aortic valve. Panel **e**: The right disc of the device was then deployed by pushing the device cable. Ao: aorta; LV: left ventricle; RV: right ventricle

Two types of surgical incisions were used to facilitate the procedure: A.3 cm-4 cm lower partial median sternotomy; B.2 cm-3 cm left anterior mini-thoracotomy. Free wall of the right ventricle (RV) was then exposed and systemic heparinization (125U/kg) was achieved. The surgeon identified the location of the puncture site by indenting the RV free wall with his index finger under the guidance of TEE, until a position perpendicular to the plane of the VSD was found. A pledgetted 4–0 polypropylene mattress sutures was placed around the chosen puncture site, and a 20-G needle was inserted into the RV cavity. A flexible 0.035-inch guide wire was introduced 4-6 cm into the RV through the needle and maneuvered into the left ventricle (LV) through the defect, the needle was then removed. A double-lumen delivery sheath was advanced about 3–5 cm into the heart over the guide wire under guidance of TEE, the inner sheath was then removed. During this process, tip of the delivery sheath as well as the guide wire should be continuously visualized through TEE in order to avoid damage of intra-cardiac tissue. Then the occluder device was delivered through the prepared loading sheath and the delivery sheath. The left ventricular disc was deployed first by pushing the cable forward while holding the delivery sheath still. Once the left ventricular disc and marker of eccentric side were visualized by TEE (Marker on the device indicate the eccentric side of the left disc), the occluder was rotated until the eccentric side of the disc was opposite to the aortic valve while the short side of the disc was located just beneath the inferior margin of the right coronary cusp of the aortic valve. After deployment of the left disc, the cable and delivery sheath were simultaneously withdrawn slowly until the left disc was snugly against the left side of the ventricular septum, then the waist as well as the RV disc were deployed. In order to avoid potential damage to the aortic valve, it was crucial to be very gentle, steady and cautious during the entire releasing phase of the device. TEE was then used to assess the presence of any undesirable changes of the aortic morphology, left ventricular outflow track obstruction, residual shunt, device mal-position as well as device-induced new regurgitation of the valves. Fluoroscopy was also performed to evaluate the device position if necessary. Then the delivery system was removed, the mattress suture tied and heparin neutralized. All patients received low dose oral aspirin (3 mg/kg/day), starting on the first post-operative day for six months.

### Follow-up

TTE was performed in second post-operative day, the day before discharge and during the follow-up. The position and stability of the device, residual shunt or valve regurgitation, particularly the aortic valve, were carefully checked during the examination. Procedure-induced valve complications were defined as new-onset or aggravation of preexisting valve regurgitation after the procedure. Twelve-lead ECG was routinely obtained on the second and third post-operative days, the day before discharge as well as the follow-up.

### Statistics

Data for nominal variables were expressed as percentages and mean ± SD or median (range) for continuous variables. The SPSS 16.0 for windows (SPSS Inc, Chicago, IL) was used for statistical analysis. Procedure failure or complications such as, residual shunt, procedure-induced aortic regurgitation (AR)/pulmonary regurgitation (PR), as well as complete atrioventricular block (cAVB), complete right bundle branch block (RBBB) were all analyzed as dependent outcome variables. Age, weight, VSD size, device size, ratio of VSD size to patient’s weight, ratio of the device size to the patient’s weight, preoperative aortic valve regurgitation or prolapse were analyzed as independent variables. Univariate analysis was performed using Student *t* test and chi-square test or Fisher exact test. Multiple logistic regression analysis was performed to study risk factors for procedure failure and the occurrence of complications. Independent variables with a *P* value less than 0.2 in the univariate analysis and factors that were considered of clinical relevance were included in subsequent multivariable analysis, Odds Ratio and its 95 % confidence intervals (CI) were calculated. *P* < 0.05 was considered as statistical significant in multivariable analysis.

## Results

### Patient character

During study period, 120 patients with doubly committed sub-arterial-VSDs were admitted for surgical intervention during study period. 86 of them (including 54 male) met the including criteria were enrolled in this study with mean age 7.0 ± 6.7 years (range 0.6 – 37)/weight 22.4 ± 14.8 kg (range 6 – 75) and mean VSD size 4.8 ± 1.4 mm (range 3–9). Mild degree aortic valve prolapse was noted in 17 patients (20 %) before the procedure. Among them 11 patients were converted to open heart surgical repair due to obvious residual shunt >3 mm (3 patients), procedure induced more than mild degree aortic regurgitation (5 patients) and device mal-position (3 patients) after device deployment in operation room.

Total 75 patients underwent successful perventricular device closure of doubly committed sub-arterial VSD with mean size 4.8 ± 1.5 mm (range 3–9) and mean device size 6.7 ± 1.7 mm (range 4–10). All patients finished the follow-up with mean follow-up time 4.5 ± 1.5 years. The detail characters of these patients are shown in Table 1. There were no death or major complications such as obvious residual shunt, major bleeding, device displacement or embolism, 3^rd^ degree atrioventricular (AV) block, procedure-induced moderate to severe aortic regurgitation during both hospital stay as well as follow-up.

**Table 1:** Baseline characteristics of patient who underwent successfully preventricular device closure of doubly committed sub-arterial VSD

Variables (*n* = 75)	Values
Age (year)	7.0 ± 7.0 (0.6-37)
Gender (F/M)	27/48
Weight (kg)	22.5 ± 15.6 (6–75)
VSD diameter (mm)	4.8 ± 1.5 (3–9)
Device size (mm)	6.7 ± 1.7 (4–10)
Hospital stay (days)	10 ± 2 (6–12)
Preoperative valve complication	
Aortic regurgitation (n)	13 (10 trivial/3mild) 17.3 %
Mild Prolapse of right coronary cusp (n)	10 (slight prolapse) 13.3 %
In-hospital complication	
Residual shunt (n)	11 (<2 mm) 14.7 %
Arrhythmia (n)	2 (transient sinus tachycardia) 2.7 %
Procedure induced AR (n)	16 (12trivial/4mild) 21.3 %
Procedure induced PR (n)	6 (trivial) 8 %
Follow-up complication	4.5 ± 1.5
Mean Follow-up time (years)	
Residual shunt	3 (<2 mm) 4 %
Arrhythmia	3 (Complete right bundle branch block) 4 %
Procedure induced AR	8 (7 trivial/1mild) 10.7 %
Procedure induced PR	6 (5 trivial/1mild) 8 %

### Residual shunt and arrhythmia

Small residual shunt (<2 mm in TEE) was noted in 11 patients (14.7 %) immediately after operation. Most of them resolved during early post-operative days while only 4 patients (5.3 %) still had residual shunt at discharge. During follow-up, only 3 patients (4 %) were noted to have 1.5 mm residual shunt on TEE. No serious postoperative arrhythmia such as 3^rd^ degree AV block was noted immediately after operation, at discharge and during follow-up. Transient sinus tachycardia was presented in 2 patients and resolved at discharge. Three patients developed complete right bundle branch during follow-up confirmed by 24 h-holter EKG (Table 1).

### Procedure induced valve regurgitation

Procedure-induced AR was noted in 16 patients (21.3 % - 12 trivial/4 mild degree) at discharge. During follow-up, 8 of them resolved and remaining 8 patients was still noted with procedure-induced AR (10.7 % - 7 trivial/1 mild). As for 13 patients with pre-existing AR, 7 resolved after surgery, while 6 patients remained the same throughout follow-up. Procedure induced pulmonary regurgitation was noted in 6 patients (8 % - all trivial grade), while during follow up 1 patient progressed to mild degree and 5 remained the same. All valve regurgitations stated above were asymptomatic and either trivial or mild degree. No significant disturbance of cardiac function was noted (Table 1).

### Multivariate analysis

Among 75 patients underwent successfully pre-ventricular device closure, each complication especially as for procedure-induced valve regurgitation was analyzed individually in this study. Univariate logistic analysis suggested that the procedure induced AR (total 16 patients) was associated with ratio of device size to patient’s weight (*P* = 0.027), Device size (*P* = 0.15), VSD size to patient’s weight (*P* = 0.07). Furthermore, multiple logistic regression analysis including these factors as well as other three clinically relevant factors (including VSD size, and occurrence of preoperative aortic regurgitation as well as aortic valve prolapse) still revealed that, only association between ratio of device diameter to weight and procedure-induced AR was statistically significant (OR = 12.3 with 95 % CI 1.5- 99.2, *P* = 0.019) (Table 2). In further analysis, a cut-off point of device diameter to weight ratio (Ratio = 0.4) was made according to our clinical experience, further analysis revealed that occurrence rate of procedure-induced AR was significantly higher in patients with device diameter to weigh ratio > 0.4 in compared with those with ratio ≤ 0.4 (*P* = 0.025) (Table 3). No other dependent outcome variables such as residual shunt, arrhythmia were related to any of the independent variables analyzed.

**Table 2:** Univariate and multivariate analysis of risk factor for procedure-induced aortic regurgitation (AR) in study cohort

Variables	Univariate	Multivariate	
		OR (95 % CI)	*P* Value
Age	NS	-	-
Weight	NS	-	-
VSD size	NS	-	NS
VSD size/Weight	0.07	-	NS
Device size	0.15	-	NS
Device size/Weight	0.027	12.3 (1.5-99.2)	*P* = 0.019
Aortic valve prolapse	NS	-	NS
Preoperative AR	NS	-	NS

*NS* non-statistically significant

**Table 3:** Cut-off point of device diameter to weight ratio (Ratio = 0.4) was made according to our clinical experience, occurrence rate of procedure-induced (PI)-AR was significant higher in patients with device diameter to weigh ratio > 0.4 in compared with those with ratio ≤ 0.4

Risk factor	Patient number	Without PI-AR (n)	With PI-AR (n)	Risk of PI-AR	P
Device/weight Ratio	≤0.4 (*n* = 42)	37	5	12 %	0.025
	>0.4 (*n* = 33)	22	11	33 %	

Among total 86 patients enrolled in this study, risk factor for procedure failure was also analyzed. In univariate logistic analysis, the procedure failure was associated with VSD size to patient’s weight (*P* = 0.11) and preoperative aortic valve prolapse (*P* < 0.001). In further multivariate logistic regression analysis including these two factors and other clinically relevant factors (including VSD size as well as occurrence of preoperative aortic regurgitation) still revealed that the procedure failure was associated with preoperative aortic valve prolapse (OR = 65 (95 % CI 7.5- 564.1), *P* < 0.001) (Table 4).

**Table 4:** Univariate and multivariate analysis of risk factor for procedure failure among study cohort

Variables	Univariate	Multivariate	
		OR (95 % CI)	*P* Value
Age	NS	-	-
Weight	NS	-	-
VSD size	NS	-	NS
VSD size/Weight	0.11	-	NS
Aortic valve prolapse	<0.001	65 (7.5-564.1)	*P* < 0.001
Preoperative AR	NS	-	NS

*NS* non-statistically significant

## Discussion

VSD is the most common congenital cardiac malformations [2]. Among them, doubly committed sub-arterial VSD is a unique anatomic subtype that is located just below the aortic and pulmonary valve [1, 2]. Different from other type of VSD, doubly committed sub-arterial VSD is known for low tendency for spontaneous closure (<1 %) as well as progressive aortic valve prolapse and insufficiency due to “Venturi effect” of the left to right shunt [3, 4]. Early intervention is recommended as for this type of VSD [13]. Open-heart surgery has been advocated as the gold standard for the treatment of doubly committed sub-arterial VSDs. However, this method is associated with complication due to CPB especially in pediatric patients [5–7]. In recent years, minimally invasive perventricular device closure has emerged as a potential treatment option in selected pediatric patients with doubly committed sub-arterial VSD [8–11]. Several studies with small cohort have proven the initial feasibility of this technique with encouraging short-term outcomes [8–10]. Also, in our retrospective comparison study, hybrid per-ventricular closure showed its obvious advantages of less invasiveness, better recovery, as well as similar mid-term outcome when compared with traditional open heart surgery in treating the selected patients with doubly committed sub-arterial VSD [11].

In this study, we further proved that this technique was associated with relative high successful rate (87 % among selected population) as well as low complication rate during long-term follow-up including 1) without major complication; 2) low residual shunt rate as well as 3) low occurrence rate of procedure induced valve complication. To our best knowledge, this is the largest cohort with long-term follow-up results (mean follow-up time 4.5 years) as for this technique in treating patients with doubly committed sub-arterial VSD. Different from transcatheter approach, perventricular device closure could provide direct access through right ventricular wall to the VSD that allows a perpendicular rout via right ventricular surface. It could then largely facilitate establishment of the delivery track and the precise deployment of the unique eccentric device. These advantages make this technique expend the limitation for minimally invasive treatment for patient with doubly committed sub-arterial VSD.

However, as for this new technique, there is still no evidence based indication as well as contraindication as for preoperative patient selection. Then in this study, multivariate logistic regression were done to screen the potential risk factors for procedure failure and the post-operative complication especially for the valve complication during low-term follow-up. Our results revealed that procedure failure was associated with the occurrence of preoperative aortic valve prolapse (even in mild degree). According to our experience [10, 11], prolapse could lead to under-estimation of the VSD size, increase risk of device mal-position as well as the interface between aortic valve and device which could cause procedure failure as well as procedure-induced valve complication [10, 11]. Patient with aortic valve prolapse, even in mild degree or without aortic valve insufficiency is not good candidate as for this procedure. Procedure-induced aortic valve dysfunction is another key safety factor to be considered as for this procedure due to close relationship between device and the valve. Actually, the position of the aortic cusp changes during the whole cardiac cycle, during diastolic phase, the aortic cups will be pushed below the annulus level. Although, we used a special design 0 mm superior rim VSD device, the interface between the device and the aortic cups is still unavoidable even after total endothelialization of the device. As show in our study, some patients with procedure induced AR still persistent during follow-up. The avoidance of choosing over-sized device in young and low weight patient should be emphasized. In this study, we revealed that ratio of the device diameter to patient’s weight was associated with procedure-induced AR, a cut-off point (Ratio = 0.4) was made according to our clinical experience with further statistical proven which indicated that the ratio above 0.4 was associated with significantly increased risk of aortic valve complication after the procedure. Then based on these evidences, the patient selection criteria for perventricular device closure of doubly committed sub-arterial VSD are proposed in Table 5.

**Table 5:** Recommended Indication as well as contraindication as for perventricular device closure of doubly committed sub-arterial VSD

Indications	Contraindications
Doubly committed sub-arterial VSD size <10 mm	VSD ≥ 10 mm
Without detectable aortic valve prolapse	Aortic valve prolapse (even mild degree)
Estimated Device size/Weight < 0.4	AR (mild degree or more)
Without AR (or only trivial degree)	Confirmed PH
Rule out PH and other CHD	

*PH* pulmonary hypertension, *CHD* congenital heart defect

There are several major limitations of this study. Due to limited case number, only eight-six patients were enrolled in this retrospective study which could compromise the validly of the study results. Further large scale, well-designed double blind randomized control study with long term follow-up is still necessary to validate the safety and efficacy of this minimally invasive procedure in treating patients with doubly committed sub-arterial VSD.

## Conclusion

Perventricular device closure is relatively safe and effective technique in selected patient with doubly committed sub-arterial VSD, with promising long-term follow-up results.

### Note

Written informed consent was obtained from the all the patients. A copy of the written consent is available for review by the Editor-in-Chief of this journal.

## Abbreviations

VSD: Ventricular septal defect
TTE: Transthoracic echocardiogram
TEE: Transesophageal echocardiogram
AR: Aortic regurgitation
PR: Pulmonary regurgitation
